# A systems analysis and improvement approach to optimizing syringe services programs’ delivery of HIV testing and referrals: Study protocol for a parallel-group randomized controlled trial (SAIA-SSP-HIV)

**DOI:** 10.1371/journal.pone.0319340

**Published:** 2025-02-25

**Authors:** Angela R. Bazzi, Alexis M. Roth, Christopher F. Akiba, Shelby L. Huffaker, Sheila V. Patel, Jessica Smith, Rose Laurano, Stephen Orme, Gary A. Zarkin, Antonio Morgan-Lopez, Barrot H. Lambdin

**Affiliations:** 1 Herbert Wertheim School of Public Health, University of California San Diego, La Jolla, California, United States of America; 2 Boston University School of Public Health, Boston, Massachusetts, United States of America; 3 Dornsife School of Public Health, Drexel University, Philadelphia, Pennsylvania, United States of America; 4 RTI International, Research Triangle Park, Durham, North Carolina, United States of America; Jamia Millia Islamia, INDIA

## Abstract

With changing drug supplies and associated drug consumption behaviors, HIV transmission has increased among people who inject drugs in the United States. HIV testing and referrals to effective prevention and treatment services are critical for individual and population health, yet multilevel barriers limit access to HIV testing for this population, even within syringe services programs (SSPs). In this organizational-level interrupted time series randomized controlled trial, we will assess the effectiveness and cost-effectiveness of an implementation strategy, the Systems Analysis and Improvement Approach (SAIA), in optimizing HIV testing and referrals to appropriate clinical services among U.S. SSPs. From 01/12/2023 to 01/07/2025, we will recruit a diverse sample of 32 SSPs nationally that directly provide HIV testing to participants. SSPs will be randomized to the active implementation arm (i.e., SAIA-SSP-HIV) or an implementation-as-usual arm (n = 16 organizations per arm). SAIA-SSP-HIV is a flexible, data-driven implementation strategy designed to help optimize SSPs’ delivery of HIV testing and referrals to appropriate clinical services for HIV prevention (e.g., pre-exposure prophylaxis) and treatment. In the active implementation arm, trained SAIA specialists will guide SSPs through three cyclical steps over 12 months: (1) process mapping to identify organization-specific needs, (2) cascade analysis and prioritization of areas for improvement, and (3) testing solutions through continuous quality improvement. In both arms, we will collect outcome data over 21 months (3-month lead-in period, 12-month implementation period, 6-month sustainment period). We will assess the initial and sustained effectiveness of SAIA and calculate its cost and cost-effectiveness. This trial presents a novel opportunity to test the effectiveness of an organization-level implementation strategy for optimizing the delivery of HIV screening and referrals in community settings that are frequented by an at-risk population. If successful, SAIA-SSP-HIV could be adapted for other infectious or chronic disease care cascades within SSPs.

**Trial registration:** ClinicalTrials.gov: NCT06025435.

## Introduction

Although HIV prevalence among people who inject drugs (PWID) in the United States dropped dramatically from 40% [1] in the 1990s to 7%–10% by 2018 [2,3], HIV outbreaks associated with injection drug use have increased over the past decade, causing renewed concern about a possible resurgence of HIV in this vulnerable population [4–8]. In the context of the ongoing opioid crisis, several factors may be contributing to this increased risk of HIV transmission among PWID. First, unregulated drug supplies contain rising quantities of fentanyl [9], which increases injection frequency and has been associated with receptive syringe sharing (i.e., using a used syringe after another person has used it) [10–12]. Second, widespread use of psychostimulants such as methamphetamine is likely contributing to increased sexual exposure to HIV [13]. Furthermore, PWID experience multilevel barriers to healthcare, reducing their likelihood of accessing HIV testing and evidence-based prevention and treatment services [14,15], highlighting a need to improve HIV services delivery in alternative, low-barrier settings.

Syringe services programs (SSPs) are a promising venue for increasing access to HIV testing and prevention and treatment services. PWID often prefer accessing health services in community-based SSPs, where staff represent trusted sources of health information and support and can effectively provide infectious disease prevention and treatment services (e.g., HIV and hepatitis C testing and treatment) and medications for opioid use disorder [15–18]. However, while many SSPs came into existence with a goal of addressing HIV among PWID [19], most have adapted to also focus on additional, emerging health crises (e.g., drug-related overdose deaths [20], shifting drug use patterns [21], COVID-19 [22]), likely reducing attention on and resources directed towards HIV services [22–25]. Even for SSPs that have the capacity to provide onsite HIV testing, de-prioritization of HIV among SSPs’ participants and inefficiencies within service delivery may suppress the reach of HIV screening and referrals [25]. To reinvigorate HIV services delivery and engage more PWID in these services, SSPs likely need additional support at the organizational level.

We designed a randomized controlled trial (RCT) to test the effectiveness of an evidence-based implementation strategy, the Systems Analysis and Improvement Approach (SAIA), in increasing SSPs’ provision of HIV testing and referrals to appropriate clinical services, which, depending on HIV test results, could involve prevention or treatment services (e.g., pre-exposure prophylaxis or antiretroviral therapy). SAIA is a flexible, multicomponent implementation strategy that supports frontline service providers by providing a comprehensive view of care delivery cascades, helping to identify areas for improvement, and iteratively testing approaches to improve quality of care [26]. In the context of HIV services, SAIA has been shown to improve the delivery of services for preventing mother-to-child HIV transmission in Mozambique, where it was also deemed acceptable among providers [27]. Recently, members of our research team adapted and pilot tested SAIA for improving the naloxone delivery cascade within SSPs, demonstrating its potential within this nonclinical community setting [28]. However, numerous differences exist between the delivery of HIV services in clinical settings SSPs, and between naloxone and HIV services delivery (e.g., staff capacity, resource and infrastructure availability, relative prioritization, complexity, standardization), warranting a need for additional implementation research on the SAIA strategy for increasing HIV services delivery within SSPs.

We developed SAIA-SSP-HIV to improve HIV services delivery among SSPs across the United States. As there is wide variation in SSPs’ provision of HIV services, and few SSPs can provide clinical services directly onsite, we focus our efforts on increasing HIV testing and the subsequent provision of referrals to appropriate clinical services depending on HIV test results. As SAIA-SSP-HIV moves beyond individual-level interventions to target organizational-level factors that impede or promote the delivery of HIV testing and subsequent referrals, it has the potential to reinvigorate and optimize these services within SSPs, effectively reaching larger numbers of PWID and better promoting individual and population health. This protocol paper describes the rationale and methods for the ongoing SAIA-SSP-HIV implementation trial, which was prospectively registered on September 6, 2023 (ClinicalTrials.gov: NCT06025435).

## Materials and methods

### Aims, objectives, and hypotheses

The overall aim of this study is to assess the impact of SAIA-SSP-HIV on U.S. SSPs’ delivery of HIV testing and referrals to appropriate clinical services, as compared to an implementation-as-usual (IAU) condition. The specific objectives and related hypotheses of this organizational-level interrupted time series randomized controlled implementation trial include:

1. To test the effectiveness of SAIA-SSP-HIV on improving SSPs’ delivery of HIV testing and referrals to appropriate clinical services (i.e., for HIV prevention or treatment, depending on HIV testing results). We hypothesize that, compared to the IAU arm, SSPs randomized to the SAIA-SSP-HIV arm will demonstrate significantly higher proportions of participants receiving HIV testing and referrals over a 12-month implementation period.
2. To test the sustained effectiveness of SAIA-SSP-HIV on improving SSPs’ delivery of HIV testing and referrals to appropriate clinical services. We hypothesize that, compared to IAU, SSPs randomized to SAIA-SSP-HIV will sustain higher proportions of participants receiving HIV testing and referrals during a 6-month sustainment period (i.e., 6 months after the active treatment phase).
3. To estimate the costs and cost-effectiveness of SAIA-SSP-HIV on improving SSPs’ delivery of HIV testing and referrals to appropriate clinical services. We hypothesize that SAIA-SSP-HIV will be cost-effective at increasing the proportions of participants receiving HIV testing and referrals compared to SSPs randomized to IAU.

### Expected results of the research

1. To expand SSPs’ reach to underserved populations at elevated HIV risk;
2. To determine the effectiveness of the SAIA implementation strategy in addressing multilevel challenges SSPs face in delivering HIV testing and subsequent referrals; and
3. To inform the use of the SAIA strategy for optimizing other infectious disease care cascades within SSPs.

### Ethical considerations

The Institutional Review Board of the University of California, San Diego reviewed this protocol and provided a Not Human Subjects Research determination on July 13, 2022 (protocol # 804265).

### Study setting

SSPs are expanding across the United States, with 473 programs listed in the public-facing directory of the North American Syringe Exchange Network as of February 2024 [19]. SSPs offer numerous services to people who use drugs and PWID, including sterile drug consumption supplies (e.g., syringes, other drug use equipment), overdose education and naloxone distribution, and testing for HIV and other infectious diseases (e.g., hepatitis C). Some SSPs also provide case management services, basic clinical services (e.g., vaccinations, wound care), medications for opioid use disorder, and supported referrals to external health and substance use disorder treatment services [29,30]. Three decades of research show that SSPs are safe, cost-saving, and effective in reducing the transmission of HIV and other infectious diseases [29]. Although there is wide variation in the size, scope, service delivery models, and reach of SSPs, most programs offer at least some HIV testing and referral services [30]. However, similar to the healthcare system, the COVID-19 pandemic disrupted routine service delivery for many programs and presented particular challenges to the delivery of services requiring in-person interaction, such as HIV testing and subsequent referrals [30]. Combined with public health authorities’ and funders’ increasing prioritization of overdose prevention, it is unlikely that most SSPs have resumed HIV testing at pre-pandemic levels. Efforts are thus needed to identify and reduce service delivery inefficiencies to optimize HIV testing and referral services [31].

The HIV prevention and treatment cascades begin with targeted outreach efforts to identify and engage at-risk individuals in HIV testing. In the general U.S. population, it is estimated that 15.5% of the approximately 1.1 million people living with HIV/AIDS are unaware their status, which can threaten individual health and enable ongoing transmission [8]. Despite the elevated risk of acquiring HIV through injection and sexual exposures [32], given the low levels of healthcare utilization among PWID, it is unlikely that most PWID receive the annual HIV testing recommended by the U.S. Centers for Disease Control and Prevention [33]. While most SSP participants should be offered HIV testing as part of routine service delivery encounters, the methods and extent to which frontline SSP staff engage participants in HIV testing and referral services varies across organizations [34], and levels of participant uptake of HIV testing may be lower than desired [25].

As of February 2024, 71% of the 473 publicly listed U.S. SSPs were providing HIV testing or education [19]. The most common HIV testing process within SSPs and other nonclinical settings involves conducting an initial HIV test, often through point-of-care “rapid” testing. Due to limitations in staff training and certifications, space, funding, and prioritization, fewer programs utilize onsite laboratory-based testing requiring a blood draw [33,34]. Following HIV testing, most SSPs then refer participants to external organizations, especially those with which they have established relationships, for clinical services including antiretroviral medications for HIV prevention (i.e., pre-exposure prophylaxis) or treatment [30,33,35]. Some SSPs also provide referral supports including transportation, incentives, and “warm hand-offs” or introductions to nearby clinical providers, though it is unknown how consistently programs provide these supports or which supports are most successful in linking participants to clinical services [8]. Rarely, SSPs support participants with later steps in the HIV prevention and treatment cascades, including medication storage and adherence supports, depending on capacity and infrastructure availability [33]. Drop-offs and inefficiencies early in the HIV prevention and treatment cascades (e.g., testing and subsequent provision of referrals) threaten subsequent cascade steps (e.g., accessing evidence-based HIV prevention and treatment services). As such, addressing these drop-offs within SSPs will be critical to meeting the goals of the national Ending the HIV Epidemic initiative [8].

### Implementation strategy

This trial will test the impact of SAIA-SSP-HIV on SSPs’ delivery of HIV testing and subsequent referrals to appropriate clinical services. As an overview, the Systems Analysis and Improvement Approach (SAIA) is a multi-component implementation strategy designed to help organizations optimize their delivery care cascades [26]. Extensive prior work has documented SAIA steps and mapped them to discrete implementation strategies as defined by the Expert Recommendations for Implementing Change [26,36]. Briefly, SAIA is comprised of three overarching steps that may be repeated iteratively. The first step is “cascade analysis” to assess organizations’ performance across sequential outcomes within their service delivery cascades. Typically, these outcomes are summarized over periods of time (e.g., weeks, months) using a Cascade Analysis Tool (CAT) that helps visualize organizations’ care cascades, identify drop-offs and inefficiencies, and determine which steps could have the greatest potential for improvement if specific inefficiencies are addressed. The second step is “process mapping,” which involves a trained SAIA specialist working with organization staff to understand and illustrate the typical flow of participants through service delivery. The third step involves continuous quality improvement (CQI) using data from the CAT to inform which steps of the service delivery process have the most potential for improvement and developing solutions to optimize service delivery at these steps. After the team tries specific, locally identified solutions, the SAIA specialist assists staff in revisiting the CAT to evaluate the impact of the solutions and determine whether to adopt them permanently, adapt and re-evaluate solutions in another cycle, or abandon them altogether. Because SAIA is designed to be responsive to the needs of the organization, management and frontline staff should be engaged in each of these steps through regular SAIA strategy meetings facilitated by the SAIA specialist. Each of SAIA’s three steps have been adapted as follows to focus on these specific outcomes early in the HIV prevention and treatment cascades:

#### 1. The HIV-CAT.

The SAIA specialist will work with up to six SSP staff members, including leadership, management, and frontline (i.e., participant-facing) staff to interpret the HIV-CAT to better understand areas of drop-off related to the delivery of HIV testing and referrals to appropriate clinical services. Organizational needs and preferences, as well as potential magnitudes for improvement (automatically calculated within the HIV-CAT), will inform decisions about which drop-off points are prioritized as areas for optimization.

#### 2. Process mapping.

SAIA specialists will work with staff to discuss and illustrate the flow of SSP participants through HIV services within their SSP. SAIA specialists will work with staff to identify processes believed to contribute to drop-offs and inefficiencies in the cascade, discuss and select potential solutions (i.e., “micro-interventions” like reorganizing the flow of participants through the SSP, incentivizing HIV testing, or scheduling structured staff supervision), and develop plans for implementing solutions based on feasibility and potential cascade gain.

#### 3. Continuous quality improvement.

SSP staff will implement the selected solutions for a minimum of four weeks. Some solutions may take longer than others to implement due to their complexity, the length of time required for observing results, or the resources available within programs (e.g., for hiring staff for a new role compared to displaying a sign that clearly describes when and where HIV testing services are offered). SAIA specialists will convene monthly meetings with SSP staff to help them review the HIV-CAT and evaluate whether the solutions implemented produced the desired improvements. Based on these results, SSP staff may decide to adopt specific solutions permanently, adapt and re-evaluate them in another cycle, or abandon them altogether and test entirely different solutions. By repeating the process of cascade analysis, process mapping, implementation of micro-interventions, and assessment of effects on cascade performance, the SAIA specialist will guide the SSP through multiple rounds of CQI over the 12-month implementation period.

### Implementation strategy fidelity and specification

A core outcome of implementation research involves intervention fidelity, or “the extent to which an intervention is delivered as intended [37].” As a potential moderating variable in intervention research, fidelity measurement is needed to reduce the risk of a Type III error (i.e., ensuring the treatment was delivered as intended and is therefore responsible for any change in outcomes). Given that implementation strategies often represent complex behavior change efforts themselves (e.g., SAIA specialists’ meetings with SSP staff), assessing fidelity of implementation strategies is similarly and critically important in trials of implementation strategies like SAIA-SSP-HIV. In addition to implementation strategy fidelity assessment, strategy specification facilitates an understanding of what strategy activities entailed, ultimately improving replicability in subsequent research or practice settings. Implementation strategy specification should delineate the actor, action, action target, temporality, dose, implementation outcome affected, and justification [38]. For this trial, we build upon Akiba et al.’s adapted specification of SAIA, which expanded the Proctor et al. specification components to include additional components focused on implementation strategy fidelity like meeting frequency, consideration of content covered in specialist sessions, session duration, quality of interactions, and participant responsiveness (Table 1) [39]. Implementation outcomes are described in greater detail below.

**Table 1:** SAIA-SSP-HIV implementation strategy specification and fidelity.

Name it:	Systems Analysis and Improvement Approach for HIV (SAIA-SSP-HIV)
**Define it:**	Facilitate development of a quality monitoring system to conduct cyclical small tests of change led by the organizational implementation teams.
**Specify it:**	
**Actor**	External specialist works and facilitates discussion with the organization’s implementation team with regards to the SAIA-SSP-HIV process.
**Actions**	Identify gaps: Present data evaluating the SSP’s^a^ delivery of the HIV care cascade with HIV-CAT.^b^ Facilitate discussions and support the implementation team to identify and develop consensus with regards to the areas of attrition along the HIV care cascade that they would like to address. Identify causes and opportunities: a. Facilitate discussions with the implementation team to review the SSP’s service structure and draw process maps documenting the flow of participants through the HIV care cascade to understand (i) why there are drop-offs or inequities in distribution at different points (root causes of participant attrition) and (ii) what it would take to address those issues (opportunities to streamline workflows and address key points of attrition). b. Assist team in developing consensus about programmatic modifications based on their importance and feasibility. Conduct CQI:^c^ a. Support and mentor the implementation team in operationalizing programmatic modifications. b. Present follow-up data on the delivery of the HIV care cascade for the implementation team to assess changes resulting from programmatic modifications. c. Repeat above actions after conclusion of the cycle.
**Action target**	Leverage programmatic data to facilitate CQI and foster a learning climate.
**Temporality**	After training the implementation team on the SAIA process and integrating enhanced instruments to collect program data into workflows to track HIV care cascade delivery indicators.
**Fidelity**	Content: Specialist completes actions 1–3 with SSPs. Coverage: SSP staff are present during actions 1–3. Duration: Actions 1, 2, and 3 take ~ 60min each. Frequency: Specialist visits SSPs in person during months 1, 4, 7, and 10. Specialist meets SSPs virtually during all other months in active phase. Quality: Specialist forms rapport with SSP staff, flexibly attends to their unique needs, and motivates SSP staff to engaged with actions 1–3. Participant responsiveness: SSP staff like working with the specialist and like participating in actions 1–3.
**Targeted implementation outcomes**	Implementation effectiveness relative to IAU.^d^ Reach: Number of HIV tests conducted Fidelity (to delivery of the HIV care cascade): Number of referrals to appropriate clinical services for HIV prevention and treatment
**Justification**	SAIA-SSP-HIV combines a broad view of the service system with iterative improvement cycles in a user-friendly way; by leveraging SAIA-SSP-HIV, SSPs can identify fillable gaps in the delivery of the HIV care cascade and apply locally generated solutions that have a higher likelihood of leading to measurable and sustained improvements in fidelity to the cascade and penetration of HIV services.

^a^SSPs, syringe services programs; ^b^HIV-CAT, HIV cascade analysis tool; ^c^CQI, continuous quality improvement; ^d^IAU, implementation as usual. Table adapted, with permission, from Akiba et al. [39].

### Study design

**Fig 1:**
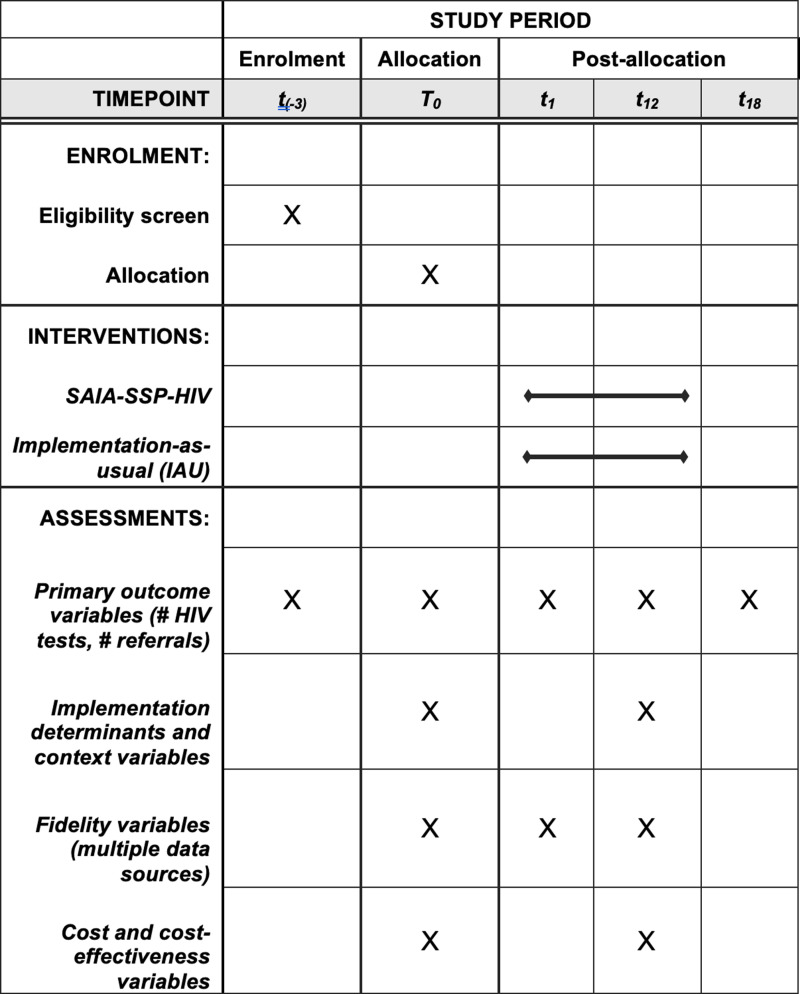
Schedule of enrolment, interventions, and assessments for the SAIA-SSP-HIV trial.

To meet our specific objectives, we will conduct a parallel-group interrupted time series RCT with 32 U.S. SSPs randomized to receive SAIA-SSP-HIV or IAU for 12 months (n = 16 organizations per arm; Fig 1). SSPs randomized to the IAU arm will not receive the active SAIA strategy or any support from SAIA specialists (i.e., IAU is characterized by the absence of SAIA-SSP-HIV).

### Recruitment and pre-randomization procedures

We will recruit SSPs from 01/12/2023 to 01/07/2025. Organizations will be considered eligible for the trial if they meet the following inclusion criteria: (1) located in a U.S. state, territory, or tribal nation, (2) operate a dedicated SSP that provides access to sterile syringes and injection equipment, and (3) have directly provided their own (i.e., not through referrals to external organizations) HIV testing services to participants within the past 30 days. SSPs will be excluded if they (1) are participating or have participated in other studies involving SAIA or its key components, or (2) intend to stop providing HIV testing services to participants within the next 21 months.

Initial recruitment efforts will be targeted towards SSPs known to our national investigator team through previous research. From there, we will leverage publicly available SSP organizational directories (e.g., the North American Syringe Exchange Network [NASEN]). Study coordinators will first email identified SSPs’ directors (e.g., executive director, chief executive officer) to briefly describe the study and request an introductory call to provide greater detail on study procedures. For SSPs expressing interest in enrolling in the study, the coordinator will set up an enrollment appointment using Zoom’s web-enabled audio and screen-sharing technology and assign a unique identification code. Recruitment will occur over 15 months, targeting two to three enrollments per month. If an SSP declines or fails to respond after three contact attempts, we will replace the organization with another potentially eligible SSP.

Following enrollment, SSPs will enter a three-month lead-in period beginning on the first day of the month they are enrolled in. During lead-in, SSPs will be asked to submit data on outcomes (HIV testing and referrals) for the month prior, disaggregated by week. Outcome data, described in greater detail below*,* will include total numbers of SSP participant encounters, HIV tests conducted, and referrals to appropriate clinical services. Outcome data will be collected using an online survey distributed automatically through REDCap, a secure online platform for managing research project surveys. Study coordinators will provide technical support to SSPs as needed to ensure timely and complete outcome data submissions.

One to two weeks prior to the end of the three-month lead-in period, we will ask organizational directors from enrolled SSPs to complete an interviewer-administered baseline survey, lasting 45–60 minutes (conducted on Zoom with the interviewer entering data into REDCap). Baseline surveys will assess programs’ internal and external characteristics, [40] implementation climate, HIV services, and operational costs.

At the end of the lead-in period, SSPs will be randomly assigned to receive SAIA-SSP-HIV or IAU using REDCap’s online randomization module. As our formative research suggests that SSPs’ HIV services delivery models vary significantly depending on organization type (i.e., non-governmental community-based organization [CBO] vs. local or regional health department or clinic), randomization will be stratified into two groups. Study coordinators will conduct randomization and deliver results to primary SSP contacts over Zoom after administering the baseline survey.

### SAIA-SSP-HIV specialist activities

We will hire and train two SAIA specialists who are knowledgeable about SSPs’ organizational cultures and service delivery models. SAIA specialist training sessions will focus on building familiarity with the specific steps of SAIA, electronic data collection instruments, and the HIV-CAT. Interactive training activities will include the provision of mock data to SAIA specialists for practicing SAIA steps. SAIA specialists will receive weekly supervision from a SAIA supervisor for the duration of the 12-month implementation period.

For SSPs randomized to SAIA-SSP-HIV, SAIA specialists will partner with leadership and frontline staff to deliver the strategy over the 12-month implementation period. SAIA specialists will meet with active implementation arm SSPs twice per month for the first three months, and once per month for the remaining nine months; these meetings will take place primarily over Zoom, with one in-person meeting each quarter. Specialists will be allotted 45 hours to deliver SAIA to each SSP (3 hours per meeting: 2 for preparation, 1 for the meeting). We chose this time allotment based on the original SAIA trial and Patel et al.’s successful naloxone pilot study [28].

During initial visits to SSPs, the assigned SAIA specialist will discuss the HIV services delivery cascade and the SAIA implementation strategy in detail with SSP leadership, observe SSPs’ processes for delivering HIV services, and review data collection procedures with attention towards identifying areas to improve documentation and streamline data collection. During subsequent visits, SAIA specialists will guide SSPs in the active implementation arm through multiple iterative cycles of cascade analysis, process mapping, and CQI, as described above. With technical support from SAIA specialists, SSPs will utilize data collection instruments developed by the study team (in collaboration with SSPs involved in our pilot study [28]) to populate the HIV-CAT.

### Data collection activities

#### Implementation determinants and context variables.

Previous implementation studies, including those utilizing SAIA, have demonstrated the importance of external context, implementation climate, and leadership engagement as determinants that influence and are influenced by quality improvement approaches [28,41]. During the initial baseline survey conducted prior to randomization, we will assess all enrolled SSPs’ basic organizational characteristics (e.g., funding sources, staff size, budget), implementation climate, and leadership engagement, which are implementation determinants we have observed in our preliminary research with SSPs [28,42]. We will measure implementation climate and leadership engagement again at 12-month follow-up for all enrolled SSPs, and once more for the SAIA-SSP-HIV arm only at 18 months, marking the end of the sustainment period.

#### Fidelity variables.

Because SAIA is delivered by a SAIA specialist, SAIA fidelity assessment will occur at the specialist level (n = 2). We will utilize a mixed-methods approach harnessing simple descriptive statistics (i.e., means, medians, standard deviations, interquartile ranges) and qualitative interviews with SAIA specialists and the staff they worked with to evaluate specialists’ fidelity to the implementation strategy. As small sample sizes represent challenges for implementation strategy fidelity assessment in general, our mixed-methods approach will utilize multiple data sources and analytic techniques to triangulate fidelity findings. Specific fidelity domains will include content, coverage, frequency, duration, quality, and participant responsiveness to SAIA, which we will capture using an encounter log in which SAIA specialists will document key information regarding session frequency and duration, SAIA steps conducted in each session, SSP staff in attendance at each session, and level of SSP staff engagement during each session [28]. This encounter log will be reviewed during weekly meetings between SAIA specialists and an experienced SAIA supervisor. Additionally, on a monthly basis, SAIA specialists will complete periodic reflections, a guided discussion tool designed to document aspects of implementation including setting, contextual and environmental factors, and adaptations to the implementation plan [43]. An additional goal of our qualitative interviews, to be conducted at the end of the trial, will be to explore any potential unintended consequences of the implementation strategy (e.g., SAIA could shift SSPs’ focus toward improving HIV services at the expense of other life-saving services).

#### Primary outcome variables.

For Objectives 1 and 2, which determine the effectiveness and sustained effectiveness of SAIA-SSP-HIV compared to IAU, our primary outcomes will include the number of HIV tests conducted and the number of referrals to appropriate clinical services for HIV prevention and treatment. As such, our primary outcomes are considered core elements of implementation effectiveness, which is a multi-faceted construct encompassing the consistency, quality, and appropriateness of an innovation’s application within an organizational setting [44]. We operationalize implementation effectiveness based on Proctor et al.’s (2011) taxonomy of implementation outcomes, with reach, measured as the number of HIV tests conducted and the number of referrals, serving as a proxy for appropriateness and consistency [45]. To operationalize these outcomes, each month SSPs will receive automated electronic forms (via REDCap) to submit data on the numbers of participant encounters, HIV tests conducted, and referrals to appropriate clinical services. SSPs will receive financial incentives every three months for timely and complete monthly data submissions [28].

#### Cost and cost-effectiveness variables.

To assess cost and cost-effectiveness of SAIA-SSP-HIV, we will utilize a modified version of the substance abuse services cost analysis program (SASCAP) [46], a validated instrument that has been applied to numerous behavioral and public health interventions involving cost and labor components [47–49]. The cost component includes questions about program costs (e.g., building and material costs), and the labor component assesses labor allocation (e.g., staff roles and salaries) across services. In addition, we will collect information on the time spent training staff and carrying out study-related activities (e.g., hours spent collecting and reporting data and meeting with SAIA specialists). Cost data will be collected monthly, and resource use questions will be integrated within the study’s fidelity and other process data collection tools. Additional cost data on building space and staff type wages will be collected at baseline and 12-month follow-up. Consistent with our other outcomes, the average cost of SAIA implementation will be assessed at the organization level.

### Data management

As detailed above, we will use REDCap, a secure online platform, for managing study data for this project. Throughout SSPs’ 21 months of enrollment, representatives of enrolled SSPs will receive monthly automated electronic forms (via REDCap) to submit outcome data on the numbers of participant encounters, HIV tests conducted, and referrals to appropriate clinical services. SSPs will receive financial incentives every three months for timely and complete monthly data submissions. One to two weeks prior to the end of the three-month lead-in period, we will also ask representatives of enrolled SSPs to complete interviewer-administered baseline surveys (via Zoom); interviewers will enter data into REDCap. To randomize programs at the end of the lead-in period, we will use REDCap’s online randomization module to randomly assign SSPs to receive SAIA-SSP-HIV or IAU. Interviewer-administered follow-up surveys will also use REDCap for data entry and management.

### Analyses and power calculations

Statistical analyses will be conducted in Stata (College Station, TX) using a single-blinded approach. Before model fitting under Objectives 1 and 2, we will assess whether there is significant variation across the three (potential) levels of aggregation for each set of key outcomes and implementation measures: (1) within-SSP level (repeated measures over time), (2) between-SSP level, and (3) SSP type (local health jurisdiction/clinic or community-based organization). We will also assess changes in outcomes over time by examining functional form. We assume that there will be piecewise linear change during the three-month lead-in period, post-implementation from baseline through 18 months, and treatment effect “deterioration” between 12 and 18 months, though non-linear functional forms may be necessary given the large number of assessments. We will also account for the total number of participant encounters for the number of HIV tests conducted outcome, and the number of HIV tests conducted for the number of referrals outcome.

For Level 1 (within-SSP), we will estimate three random effects as growth over time. On the basis of the structure of the time steps with *α*_pis_, we will estimate a random intercept (*π*_*0is*_), which is the estimated (conditional) mean value of the outcome at time =  0 (e.g., run-in baseline) and two random slopes (*π*_*1is*_*, π*_*2is*_): (1) the estimate per-year change in Y from the run-in phase through 18 months and (2) the “deterioration” phase, capturing how much reduction there is in change over the last year, if any, for the SAIA arm. These values will vary across SSP *i* within county *s*. In Level 2 (between-SSP), the Level-1 intercept and slopes will be the outcomes at Level 2.

*Β*_*pqs*_ is a matrix of coefficients corresponding to the effect of between-individual predictor *q* on Level-1 outcome *p* (intercept, slope) within SSP *s*. The key predictor included in *χ*_*qis*_ is a 0/1 dummy indicator indicating whether SSP *i* was in the SAIA condition or IAU. *Β*_*1s*_ will capture the average change over time in HIV testing and referrals to appropriate clinical services for HIV prevention and treatment, which (1) should not be significantly different from zero during the lead-in period, and (2) will vary by SAIA and IAU after implementation (by virtue of the SAIA x time period 1 interaction). *Β*_*0s*_ will capture the conditional mean level of study outcomes, which, if it varies by treatment condition, would capture a mean shift in our study outcomes. *Β*_*2s*_ will capture the change over time in study outcomes during the sustainment period; if it interacts with the SAIA indicator, it will capture slope differences over time beyond 1 year between SAIA and the IAU condition, with Year 1 gains maintained if this parameter estimate is not significant. Level 3 will capture SSP type-level variability in intercepts and, if necessary, slopes over time.

The main analyses will be modeled under full information maximum likelihood and supplemented by multiple imputation for missing data on key predictors for models under Objectives 1 and 2. Both produce accurate estimates and standard errors under the assumption that missingness is predictable by variables that are observed but unrelated to the values that are missing themselves (i.e., missing-at-random). Results from outcomes analyses across 20 multiply-imputed datasets are then combined, accounting for both between-imputation and within-imputation variation. In assessing sensitivity to the missing-at-random assumption, we may explore latent pattern mixture models for non-ignorable missingness. These methods have been utilized in other studies conducted by members of the research team.

We structured a Monte Carlo power analysis for Objectives 1 and 2 by structuring a population model reflecting weekly assessments for a 3-month lead-in period, a 12-month active intervention phase, and a 6-month sustainment period. The mean and standard deviation of the number of people who test for HIV in the target SSPs was 7.32 (2.94); these values were used as the run-in period mean and SD in the simulation. Effect sizes from a pilot study that used a similar SAIA platform ranged from.48 to 1.50; thus, we power the current study in anticipation of large effect sizes (d = .8). The mean shift in post-implementation outcomes favoring SAIA that corresponds to an effect size of.8 would be an additional 2.35 people tested under SAIA. The per-week increase in number tested under SAIA to reach a longitudinal Cohen’s d effect size of.8 by 11 weeks would be an increase of.21 persons per week. We generated 250 synthetic samples of n = 32 and analyzed the samples in Mplus while imposing (a) the random effect structure from pilot estimates and (b) a state-level ICC of.05. Power to detect a post-implementation mean shift and differences in slopes over time were greater than 80%. Detecting treatment effect deterioration from months 1–12 to month 13–18 for power of.80 requires deterioration equivalent to a Cohen’s d of.48; despite a sample size of 32, the extremely large number of repeated measures offsets the sample size with regard to statistical power to detect slope differences.

We will calculate cost estimates for each SAIA step at each SSP. For each step we will multiply the quantity of the resource (e.g., labor) used by its price (e.g., wage), then calculate the average cost for each step and all five steps for an SSP. Our effectiveness measures for the cost-effectiveness analysis (CEA) are the number of HIV tests conducted (H3.1) and the number of referrals to appropriate clinical services for HIV prevention and treatment (H3.2). Following the Second Panel of Cost Effectiveness in Health and Medicine’s recommendations, we will combine the cost estimates with the estimated change in outcomes, such as the average number of HIV tests conducted at 12 and 18 months. For CEA, we will compare cost and outcomes from the two study arms, listing each arm in order of increasing cost; we expect the IAU arm will be lower cost and listed first. To derive the cost-effectiveness ratios, we will calculate the difference in costs and outcomes between the two arms. The incremental cost-effectiveness ratio (ICER) will then be calculated as the ratio of the difference in costs to the difference in outcomes (e.g., if effectiveness is defined as the number of referrals to appropriate clinical services for HIV prevention and treatment, the ICER represents the incremental costs per additional referral). We will calculate cost-effectiveness acceptability curves (CEACs) that incorporate the inherent joint variability of the cost and effectiveness estimates and show the probability that the strategy is cost-effective as a function of the policymaker’s intrinsic valuation or willingness to pay for the outcome. Non-parametric bootstrap methods will be used to calculate CEACs.

## Discussion

HIV transmission can be effectively mitigated by identifying and engaging at-risk populations in testing and providing referrals to appropriate clinical prevention and treatment services [50]. Scaling up the delivery of these services to PWID is especially critical at a time when changes in the unregulated drug supply and associated behaviors place this historically underserved population at increased risk of infection [2–8]. SSPs, though they vary in size and scope, are uniquely poised to reach at-risk PWID, and the SAIA-SSP-HIV trial presents a novel opportunity to test the effectiveness of an organization-level implementation strategy for optimizing the delivery of HIV testing and referrals within this service setting. If successful, SAIA-SSP-HIV could be used to scale up HIV testing and service referrals among hundreds of SSPs operating across the United States, ultimately bringing the nation closer to meeting its goal of Ending the HIV Epidemic [8,51]. Findings from this trial could also inform research and programmatic efforts to scale up point-of-care testing and subsequent service referrals for other infections that cause significant morbidity and mortality among PWID such as hepatitis C, which has been prioritized for prevention efforts nationally and globally [52].

There are limitations of this study design, including a lack of individual-level outcomes (precluding, for example, analyses focused on equity in service delivery) and an exclusion of organizations that are not yet providing any HIV testing services. Distinct study designs, data collection systems, and implementation strategies could help to answer research questions about individual-level service utilization and health outcomes, or ways to best support SSPs in initiating HIV testing and referral services for the first time (which current estimates suggest comprise the minority of U.S. SSPs [30]). We also acknowledge, as another limitation, that SSPs face many challenges with service implementation that SAIA-SSP-HIV may not be able to address, including staffing and funding constraints and competing priorities of frontline staff members, leadership, and participants. However, a strength of the SAIA implementation strategy is its flexibility in tailoring goals and activities to organizations’ unique contexts through individualized coaching, which may enable organizations to achieve some service delivery improvements despite specific local constraints.

Despite these limitations, the SAIA-SSP-HIV trial, to our knowledge, represents the first RCT of an implementation strategy focused on improving an infectious disease care cascade within SSPs. It builds upon a prior pilot study suggesting the potential effectiveness of SAIA for increasing naloxone distribution through two SSPs, which resulted in a 105% average increase in the number of doses of naloxone distributed per week following initiation of the SAIA implementation strategy [28]. However, delivering HIV services in non-clinical settings like SSPs involves a distinct care cascade conceptualization, warranting additional testing through a sufficiently powered implementation trial to detect effectiveness of SAIA-SSP-HIV. Additionally, because SSPs’ operational structures vary substantially across U.S. states and local jurisdictions, this study also seeks to expand the translatability of SAIA by exploring implementation outcomes among diverse SSPs located across the country.

In conclusion, SAIA-SSP-HIV could help optimize the delivery of HIV testing and referrals in community settings that are frequented by a vulnerable, at-risk population. As the first implementation trial focused on an infectious disease care cascade within SSPs, findings from the SAIA-SSP-HIV trial will shed light on how to support non-clinical settings in optimizing delivery of screening services and subsequent referrals into clinical care. If effective, SAIA-SSP-HIV could potentially be scaled up in support of key Ending the HIV Epidemic initiatives [8,51] and could possibly be adapted for optimizing other care cascades in SSPs and other community settings.

## Supporting information

S1 File SPIRIT checklist. (DOCX)

S2 File StaRI checklist. (DOCX)

S3 File IRB protocol. (PDF)
