# Supplementary material for: A systems analysis and improvement approach to optimizing syringe services programs’ delivery of HIV testing and referrals: Study protocol for a parallel-group randomized controlled trial (SAIA-SSP-HIV)
Source: PLoS One. 2025 Feb 25;20(2):e0319340. doi: 10.1371/journal.pone.0319340 (PMC11856318; doi:10.1371/journal.pone.0319340)
Supplement: S3 File — (PDF) [file pone.0319340.s003.pdf]

#804265 - SAIA-PrEP: a systems analysis and improvement approach to optimize PrEP implementation in syringe service programs

Protocol Information

Submission Type	Review Type	Status	Time in Current Status
**New**	**Not Human Subjects Research**	**Not Human Subjects Research**	**Since July 13 – 2 years**

Feedback

NHSR Determination Date

Jul 13, 2022

NHSR Determination Comment

The above referenced project has been reviewed by the Director of the UCSD HRPP, IRB Chair, or IRB Chair's designee and is certified as non human subjects research according to the Code of Federal Regulations, Title 45, part 46 and UCSD Standard Operating Policies and Procedures; and therefore, does not require IRB review.

Though certified as not human subjects research, the investigator should ensure that the activities associated with the project are conducted in compliance with applicable UCSD and Rady Children's Hospital – San Diego policies and ethical standards as well as local, state, and federal regulations.

This determination does not exempt the PI and study team from their responsibilities under UC San Diego PPM 100-5 (Responsibilities section, item d) or from any other approvals or

permissions required by applicable laws or university policies.

The protocol listed the following funding (or potential funding) information: Federal, NATIONAL INSTITUTE OF DRUG ABUSE

Best wishes for the successful conduct of the project.

Project Basics

STUDY TITLE

SAIA-PrEP: a systems analysis and improvement approach to optimize PrEP implementation in syringe service programs

PRINCIPAL INVESTIGATOR

Bazzi,Angela

Lead Department:

School of Public Health

Facesheet Inclusion

General Information

SUBMISSION TYPE

Administrative Determination or Registration

Submission for Administrative Determination or Registration.

Activity Not Human Subjects Research

PI is a PI-eligible UCSD employee

Yes

LOCATION WHERE ACTIVITY(IES) WILL BE PERFORMED

UCSD Facilities or Sites (e.g., school, hospital or clinics, etc.)
Off-Campus (In California)
Off-Campus (In USA outside of CA)
Other (specify below)

Enter other research activity location

Research activities will occur at 32 syringe services programs (SSPs) throughout the United States, and at RTI, International and Drexel University, the other two institutions collaborating on this project (where the other investigators are based).

LAY LANGUAGE SYNOPSIS OF THE PROPOSED ACTIVITY

For the first time in decades, HIV incidence is increasing among people who inject drugs (PWID). Pre-exposure prophylaxis (PrEP) is a safe, effective, evidence-based HIV prevention strategy recommended for at-risk PWID. However, despite the acceptability of PrEP among PWID, access remains low, and uptake has lagged far behind that observed in other vulnerable populations. Delivering PrEP to PWID through syringe services programs (SSPs), a trusted and well-utilized institution, is acceptable and feasible, yet interventions are needed to support and scale-up the implementation of these services. This randomized controlled trial uses an interrupted time series design to test whether an evidence-based, organization-level intervention, the Systems Analysis and Improvement Approach (SAIA), can improve the delivery of PrEP services among 32 SSPs located in Ending the HIV Epidemic priority jurisdictions. Specific aims include assessing the initial effectiveness of SAIA after 12 months (Aim 1), sustained impacts at 24 months (Aim 2), and cost-effectiveness (Aim 3). To our knowledge, this will be the first RCT of an organizational-level intervention to optimize the SSP-based PrEP delivery cascade. If successful, SAIA could be disseminated to the ≥430 SSPs nationally and in global settings, carrying the potential for exceptional impact amidst

persistent HIV transmission in PWID.

Study Personnel

Update the PI line by pressing the Edit Pencil and answer the pop-up questions Do not list any other personnel here. If needed, add an administrative contact in Permissions.

Person

Bazzi,Angela

Home Unit

No Item Selected

Researcher Role

Principal Investigator

Permissions

Full Access

Person

Bazzi, Angela

Home Unit

No Item Selected

Researcher Role

Permissions

Full Access

Person

VALASEK, CHAD

Home Unit

School of Public Health

Researcher Role

Key Person

Permissions

Full Access

Do any of the personnel listed above have any potential conflict of interest related to the research?

No

Funding

Choose the option that describes the funding for this project.

Funding to UCSD current or pending in Proposal Development, IP or Award stage (Department or Fiscal contact has submitted a Proposal, IP or Award record in the Grants Module)

Type of Funding

Federal

[illegible]

FUNDING INFORMATION
Federal
Health & Human Services (e.g., PHS, NIH, CDC, FDA, CMS)

If your project is pending please search for your Institutional Proposal and copy and paste the IP number into the search below.
If your project is active please search for your Award and copy and paste the Award ID into the search below.

Institutional Proposal #30145755: SAIA-PrEP: a systems analysis and improvement approach to optimize PrEP implementation in syringe service programs

Principal Investigator: Angela Bazzi
Sponsor: DRUG ABUSE, NATIONAL INSTITUTE OF
Sponsor Type: Federal
Prime Sponsor:
Prime Sponsor Type:

Not Human Subjects Research

Does the activity constitute a clinical investigation under FDA regulations (e.g., testing an In Vitro Diagnostic device, medical device, or drug/ biological product)?
No

Is this activity solely designed as a *Evidence-Based Practice/Quality Improvement/Quality Assurance* project?

All statements below must be TRUE to answer Yes:

- Implementing the practice as outlined in the project **will not incur patient harm.**
- The practice change outlined in the project is **not new or novel** and **has been published elsewhere.**
- The practice outlined in the project will be **implemented in a practice location.**
- **All staff and affected patients** in the project location will be **expected to participate** in the project.
- The project is **not testing issues or adding research questions that go beyond common practice.**
- The project will **not randomize patients** into different intervention groups.
- The project will **not deliberately delay interpretation of data.**
- [illegible]

- The project will **not deliberately delay or abbreviate feedback** to those who would benefit from the findings to enhance likelihood of publication.
- The project has **no funding support from an outside organization with a commercial interest in the use of the results.**

Yes

Do the proposed activities involve a systematic investigation, including research development, testing and evaluation?

Yes

The proposed activity as described does not constitute human subjects research. IRB review is not required.

Confirmation by the Office of IRB Administration is not required. We recommend that you print this form for your records.

If you prefer confirmation from the Office of IRB Administration, please submit this form along with supporting documentation (e.g., project description).

Confirm the following statements:

- The information provided in this application is complete and accurate to the best of your knowledge, and that you agree to conduct the project in compliance UCSD and Rady Children's Hospital, San Diego policies as well as state and federal regulations.
- If the activities conducted as part of this project change in the future whereby the project meets the definition of human subjects research, you will submit an IRB application for review and approval prior to conducting human subjects research activities.

I confirm that the preceding statements are true.

Supporting Information

Supporting Document

1_SAIA-PREP PROTOCOL_UCSD SBS TEMPLATE_REVISED_070822.DOC

Attachment Type

Protocol

Name/Version

Supporting Document

4_SAIA-PREP ASSESMENTS BASELINE FOLLOWUP.DOCX

Attachment Type

Data Collection Instruments

Name/Version

Supporting Document

6_SAIA-PREP_INTERVENTION_DETAILED_PROCEDURES.DOCX

Attachment Type

Other

Name/Version

Details on the Intervention

Supporting Document

7_SAIA-PREP ASSESMENTS 3MO RUN-IN PERIOD.DOCX

Attachment Type

Data Collection Instruments

Name/Version

Added Information - Optional

Include any additional information that you want to communicate about the study.

Assurance/Acknowledgement

By submitting this form, I confirm that the information within this form is accurate and complete.

I am the Principal Investigator

Administrative Details Form

Determinations

Review Type

Study Status

UCSD Human Research Protections Program
New Social and Behavioral Sciences Application
RESEARCH PLAN
Version Date 10/01/09
(Enter text in the **white space areas** below each numbered heading bar. **Expand the size of table cells as needed** – to multiple pages if needed.
(See SBS Application Instructions for explanation of headings and information to be provided)

1. PROJECT TITLE

SAIA-PrEP: a systems analysis and improvement approach to optimize PrEP implementation in syringe service programs

2. PRINCIPAL INVESTIGATOR, FACULTY ADVISOR, SUPERVISOR

Angela Bazzi, PhD, MPH (MPI; Contact PI); Barrot Lambdin, PhD and Alexis Roth, PhD (MPIs)

3. FACILITIES

This study will be conducted in professional offices of UCSD investigators' offices (for phone and virtual interviews) and of participants (syringe service programs [SSPs] nationally).

4. ESTIMATED DURATION OF THE STUDY

4 years.

5. SPECIFIC AIMS (2 paragraphs maximum)

For the first time in decades, HIV incidence is increasing among people who inject drugs (PWID). Pre-exposure prophylaxis (PrEP) is a safe, effective, evidence-based HIV prevention strategy recommended for at-risk PWID. However, despite the acceptability of PrEP among PWID, access remains low, and uptake has lagged far behind that observed in other vulnerable populations. Delivering PrEP to PWID through syringe services programs (SSPs), a trusted and well-utilized institution, is acceptable and feasible, yet interventions are needed to support and scale-up the implementation of these services. This randomized controlled trial uses an interrupted time series design to test whether an evidence-based, organization-level intervention, the Systems Analysis and Improvement Approach (SAIA), can improve the delivery of PrEP services among 32 SSPs located in Ending the HIV Epidemic priority jurisdictions.

Specific aims include: assessing the initial effectiveness of SAIA after 12 months (Aim 1), sustained impacts at 24 months (Aim 2), and cost-effectiveness (Aim 3). To our knowledge, this will be the first RCT of an organizational-level intervention to optimize the SSP-based PrEP delivery cascade. If successful, SAIA could be disseminated to the ≥430 SSPs nationally and in global settings, carrying the potential for exceptional impact amidst persistent HIV transmission in PWID.

6. BACKGROUND AND SIGNIFICANCE (2-3 paragraphs maximum)

The recent saturation of illicitly manufactured fentanyl[1] and psychostimulants (e.g., methamphetamine) in U.S. drug markets has been associated with HIV outbreaks in diverse regions of the country, including Ending the HIV Epidemic jurisdictions[2] with longstanding SSPs (e.g., King County, WA; Suffolk County, MA).[3,4] In fact, for the first time in two decades, an increase in HIV incidence has been detected among PWID nationally,[5,6] suggesting that this population would benefit from improved access to PrEP, an evidenced-based intervention that is recommended for preventing HIV transmission in contexts of sexual- and injection-related exposures.[7]

Despite the effectiveness and acceptability of PrEP among at-risk PWID,[7,8] access to this evidence-based HIV prevention strategy remains low,[9,10] due to social and structural barriers (including addition-related stigma and poverty) that limit utilization of healthcare services delivered within traditional clinical settings.[11,12] However, many key components of the PrEP delivery cascade, including PrEP education, HIV testing, and linkage to onsite and external medical services, can be offered outside of clinical settings and are already being provided by some SSPs involved in our preliminary studies.[13,14] SSPs are highly trusted by PWID and effectively engage some of the most socially and structurally marginalized members of this population in prevention services.[11,15-17] Thus, SSPs are well-positioned to increase PrEP access for PWID.

The Systems Analysis and Improvement Approach (SAIA) is an evidence-based, multicomponent implementation strategy for improving the delivery of HIV prevention services.[18-20] It involves five cyclical steps including (1)

analyzing service delivery data to identify priority areas for system improvements, (2) mapping processes and building consensus around programmatic modifications to address priority areas, (3) implementing programmatic modifications, (4) assessing effects of programmatic modifications on improving delivery of services across the cascade, and (5) repeating steps 1-4 as needed. Although SAIA has been translated to improve SSPs' delivery of harm-reduction services such as overdose education and naloxone distribution,[21] it has yet to be tested with PrEP. The scientific premise of this RCT is that SAIA will effectively boost and extend the PrEP delivery cascade within SSPs assigned to the SAIA intervention condition (relative to treatment as usual). This trial will be the first to test whether an organizational-level intervention can optimize the PrEP delivery cascade within SSPs.

7. PROGRESS REPORT/PRELIMINARY STUDIES

Our investigative team possesses highly relevant expertise and preliminary data from studies demonstrating PrEP need, acceptability, and facilitators of access among PWID;[22-27] the feasibility and acceptability of PrEP implementation within SSPs;[14,28] and the effectiveness and cost-effectiveness of SAIA in improving SSPs' naloxone delivery cascade.[21] These studies support the feasibility of this innovative proposal to improve the SSP-based PrEP delivery cascade, which, if successful, could be disseminated to the ≥430 SSPs operating nationally,[29] carrying potential for exceptional impact amidst persistent vulnerability to HIV transmission in this population.[30,31]

Our multidisciplinary investigative team is eminently qualified to carry out the proposed research. The MPIs possess complimentary expertise in HIV, PrEP interventions, implementation science research with PWID and SSPs, and the SAIA approach. **MPI Angela R. Bazzi, PhD, MPH**, Associate Professor at University of California San Diego's Herbert Wertheim School of Public, is an infectious disease epidemiologist with expertise engaging PWID and SSP staff in mixed-methods research on the acceptability of and barriers to accessing PrEP (K01DA043412).[11,13,22,23,27,32] She is currently leading an RCT testing the efficacy of an individual-level behavioral intervention to increase PrEP uptake and adherence among PWID in MA (R01DA051849). For this proposal, she will contribute to the overall study design and protocols, ensure that intervention content is responsive to the needs of SSP clients, oversee outcome data collection, and serve as Contact PI. **MPI Alexis M. Roth, PhD, MPH**, Associate Professor at Drexel University's Dornsife School of Public Health, is a social and behavioral scientist with a strong track record leading research to improve PWID health outcomes by co-locating prevention services (including PrEP) within low-barrier and trusted institution, including SSPs.[14,33,34] Dr. Roth led the first and only PrEP demonstration project for women who inject drugs in the United States. Her research demonstrates the feasibility and acceptability of delivering PrEP in this context (R21DA043417)[35,36] while also identifying important PrEP implementation challenges.[28,37,38] Based on this expertise, she will lead the development of intervention protocols, train SAIA-PrEP specialists, oversee intervention fidelity, and provide ongoing support and training when implementation challenges arise. **MPI Barrot H. Lambdin, PhD,** senior implementation scientist at RTI International, has led numerous implementation science initiatives to address disparities in access to evidence-based interventions for populations at risk of HIV acquisition in the United States and globally.[39-49] He leads studies focused on improving naloxone distribution in SSPs, including an RCT testing whether external facilitation improves SSPs' adherence to 20 best practices for naloxone distribution (successfully enrolling 104 SSPs nationally), national surveys of SSPs (achieving 75% or higher response rates), and a successful pilot of SAIA for improving naloxone delivery in SSPs (R21DA046703, R01DA046867). He will lead the quantitative and health economics teams analyzing data for this proposal. Our team will be supported by **Co-I Gary Zarkin, PhD**, an RTI-based economist with expertise carrying out NIDA-funded cost, cost-effectiveness, and cost-benefit analyses of data from intervention RCTs with substance-using populations, including a recent study that was highlighted as a template for cost-effectiveness methods in the final report of the Second Panel on Cost-Effectiveness in Health and Medicine.[50] He co-leads NIDA's data coordinating center for the HEALing Communities Study (UM1DA049394). **Co-I Antonio A. Morgan-Lopez, PhD,** is an RTI-based statistician with >20 years of experience developing and applying quantitative methods in substance use prevention and treatment research, including organization-level RCTs. He has been PI and/or lead statistician for group-randomized RCTs, [51-53] trials with non-compliance and systematic dropout, [54-57] and multilevel studies with quasi-experimental designs.[58,59]

8. RESEARCH DESIGN AND METHODS (1 page maximum)

To meet our study aims, we will conduct an interrupted time series RCT with 32 SSPs recruited from Ending the

HIV Epidemic priority jurisdictions.[2] Each SSP (organization) will be randomly assigned to the SAIA intervention for 12 months (n=16 SSPs) or treatment as usual (n=16 SSPs). Outcomes include the proportion of eligible SSP clients (e.g., HIV negative with PrEP indication) who receive (1) PrEP education, (2) HIV testing, (3) PrEP linkage (to onsite or external PrEP services), and (4) verification of PrEP initiation (with follow-up supports as needed). Using an electronic data portal provided by the study, outcomes will be collected during a 3-month run-in period and then over the course of 24 months, allowing us to identify the immediate impacts of SAIA at 12 months (Aim 1) and sustained impacts at 24 months (Aim 2). We will also use an activity-based costing approach to assess the cost and cost-effectiveness of SAIA compared to treatment as usual (Aim 3).

Data Collection: Outcome data used to assess the impact of SAIA will be collected electronically from all SSPs (i.e., in both trial arms) during the 3-month run-in period and for 24 months following randomization. In addition, we will conduct surveys at baseline, 12, and 24 months post-randomization. ***Data collection on outcomes in 3-month run-in period:*** Before randomization, there will be a 3-month run-in period during which SSPs will provide outcome data monthly (i.e., on total numbers of SSP clients and numbers receiving PrEP education, HIV testing, PrEP linkage, and PrEP initiation verification). While we request data uploads monthly, we will also request that SSPs disaggregate their data for each week within the month. Weekly data will increase the sensitivity of our study outcomes to SAIA and increase the power of our study. ***Baseline survey and stratified randomization:*** Following the 3-month run-in period of outcome data collection, the study coordinator will administer the baseline survey followed by the randomization protocol. The baseline survey will assess organizations' internal and external characteristics, including size, budget, numbers of staff and volunteers, environment (urban/rural), and type (governmental vs. non-governmental). SSP location (urban or rural) and type (governmental vs. non-governmental) will be used to create four strata to carry out our randomization procedures. Within each stratum, enrolled SSPs will be allocated in a 1:1 ratio to the two study arms, with equivalent proportions of SSPs in each. ***Baseline, 12-, and 24-month follow-up surveys:*** Additional SSP-specific data will be collected at baseline, 12, and 24 months after randomization from all enrolled sites. The designated primary contacts at SSPs will complete the surveys. Items will assess SSPs' organizational characteristics (e.g., location, number of staff, budget) and general information about types of staff involved with PrEP-related service delivery (e.g., roles, key services provided by each). This survey, which takes 30–45 minutes to complete, will be administered at baseline and 12 months and 24 months post-randomization to capture any potential changes over time. Participating SSPs will receive $100 compensation for their participation in each survey, for a total of $300.

Data Analyses: Statistical analyses for Aims 1 and 2 will be conducted by Co-I Morgan-Lopez, who will be blinded to randomization assignments, using Stata v16.1 (College Station, TX). Our approaches for conducting multilevel linear modelling is as follows: Before model fitting under Aims 1 and 2, we will assess whether there is significant variation across the three (potential) levels of aggregation for each set of key outcomes and implementation measures: (1) within-syringe SSP level (repeated measures over time), (2) between-SSP level, and (3) state level. We will also examine the functional form of changes over time in outcomes. For now, we assume that piecewise linear (i.e., linear change during the 3-month run-in period, post-implementation linear change from baseline through 24 months, and a period of treatment effect "deterioration" [if any] between 12 and 24 months) will be the predominant functional form, though with this large number of assessments, nonlinear forms may be necessary.[55,57,60] ***Cost and cost-effectiveness analyses (Aim 3):*** We will implement an activity-based costing approach that identifies and assigns dollar values to the time spent by SSP personnel implementing the SAIA intervention. To achieve Aim 3, we propose a rigorous costing approach, including a cost and cost-effectiveness analysis of SAIA to improve the PrEP delivery cascade at SSPs. Our approach entails working with each SSP in the RCT, the study team, and the SAIA specialists. Working closely with these stakeholders will be critical to collecting cost data as SAIA is implemented to ensure accurate and complete data. Under Co- I Zarkin's leadership, the cost team will adapt and develop economic data collection tools, collect economic data, estimate the cost of each SAIA intervention step, and perform a cost-effectiveness analysis. We follow the Second Panel on Cost Effectiveness in Health and Medicine's recommendations.[61,62]

9. HUMAN SUBJECTS (2 paragraphs maximum)

Eligibility for SSPs (organizations) included in this study are: (1) being an SSP (defined as an organization that provides access to and disposal of sterile syringes and injection equipment for PWID[63]) listed in the North American Syringe Exchange Network online directory;[29] (2) being located in an Ending the HIV Epidemic priority jurisdiction;[2] and (3) having provided HIV testing to clients in the last 30 days (~88% of SSPs). We focus on SSPs currently offering HIV testing because it is an essential component of the PrEP delivery cascade, and SAIA focuses on improving ongoing implementation. SSPs participating in MPI Bazzi and Roth's ongoing R01s (n=3) will be excluded; MPI Lambdin's RCT with SSPs is now nearing completion and thus will not pose a contamination risk to this proposed study. SSPs will be screened for eligibility during an introductory call between the Study Coordinator and the SSPs' organizational directors, a process that will not pose risk to human subjects, as the eligibility criteria are associated with their workplaces (not themselves as individuals).

10. RECRUITMENT

We will recruit SSPs into the RCT using MPI Lambdin's successful approach that has yielded 76%–78% participation in cross-sectional studies and enrollment of 104 SSPs into a national, longitudinal RCT.[63-67] First, using contact information from the publicly available electronic directory of the North American Syringe Exchange Network,[29] our study coordinator will email SSPs' directors (e.g., executive director, chief executive officer) to briefly describe the study and request an introductory call to provide greater detail on study procedures. When SSPs agree to enroll in the study, the coordinator will set up an enrollment appointment using Zoom's web-enabled audio and screen-sharing technology. Recruitment will occur over 12 months, targeting two to three enrollments per month. If an SSP declines or fails to respond after three attempts to contact them, we will replace them with the next randomly selected SSP from the pool of eligible SSPs. Based on our experience and preliminary data, of the 120 SSPs in Ending the HIV Epidemic priority jurisdictions,[2] we anticipate 106 (88%) will have delivered HIV testing in the past 30 days, 80 (75%) will be interested in participating, and of those, we will randomly select 32 (40%) for participation.

11. COMPENSATION FOR PARTICIPATION

This study is of an organizational-level intervention with syringe service programs (i.e., SSP sites). SSP sites will receive prorated financial incentives for the number of completed data submissions, up to $300 per SSP for complete data submissions over the 3-month run-in period ($100 per month) prior to the RCT, and then for monthly data submissions for rest of the 24-month period post randomization ($100 per month) as well as survey completion at baseline, 12, and 24 months ($100/survey). In total, SSPs will be eligible to receive $3,000 compensation for complete data submitted for the study, which will be distributed in the forms of University checks.

12. INFORMED CONSENT

Instead of obtaining informed consent, we will provide SSP (organization) leadership and their designated staff with a research information sheet explaining the study purpose and procedures and providing contact information for study staff.

13. ALTERNATIVES TO PARTICIPATION

The alternative is not to participate in the study. SSP (organization) leadership and their designated staff will be informed that they may decline participation or discontinue the study without facing any negative consequences.

14. POTENTIAL RISKS

This is a minimal-risk study. Study participants will be asked for their name, contact information, gender, and race/ethnicity. They will not be asked to share any sensitive information—the only information that they will share is information on the day-to-day operations of their programs. Thus, we do not anticipate potential risks from the sharing of sensitive information, nor do we anticipate any legal or civil liberty risks because of study participation. Potential study participants may have very busy work schedules and may feel uncomfortable participating in the study because of time constraints.

15. RISK MANAGEMENT

We will employ a number of risk management strategies, including:

- **Maintaining Participant Confidentiality:** The names and contact information of participants will be recorded in an electronic database, filed by participant ID number, and used by the Study Coordinator and SAIA-PrEP Specialists contacting SSPs for study enrollment in the RCT, survey administration, and intervention activities. The database will be password protected and kept on the Specialist's computer. The Specialist, Study Coordinator, and the Principal Investigator will be the only staff to have the password. The computers will be backed up regularly on a server and protected by security encryption software.
- **Participant ID Numbers:** For all data collection purposes, each SSP will be identified by an ID number, not by name. This number will be recorded on outcome data, surveys, and the SAIA-PrEP Specialist's database.
- **Confidentiality in Dissemination of Findings:** Participants will be assured that the information they provide will not be linked to them as individuals in any study reports, publications, or presentations.
- **Contacting Research Project Staff:** Participants are encouraged to contact a member of the project staff should questions or problems arise. Contact numbers are given on the research information sheet described in section 12 above.
- **Study Staff Training:** All study personnel (i.e., the primary investigators, interviewers, and research assistants) will be trained in good clinical practice and confidentiality protections before recruiting and data collection commence.

16. POTENTIAL BENEFITS

There are no direct benefits of participation in this study for individual participants; however, the goal of this study is to produce information that will improve PrEP uptake for HIV prevention in the vulnerable population of SSP clients and their communities, ultimately helping to improve public health outcomes.

17. RISK/BENEFIT ASSESSMENT

We judge the importance of the knowledge resulting from this study and the potential for improving PrEP uptake among SSP clients nationally to be high; thus, the anticipated societal benefits have been determined to outweigh the minimal risks associated with participation in this study.

18. QUALIFICATIONS, TRAINING, CULTURAL LITERACY AND ROLES OF THE PI AND RESEARCH TEAM

All study personnel (i.e., the primary investigators, interviewers, and research assistants) will be trained in good clinical practice and confidentiality protections before recruiting and data collection commence.

19. FUNDING FOR THIS PROJECT

NIH/NIDA Grant Number: R01DA056883.

20. CONFLICT OF INTEREST

We have no conflicts of interest to disclose.

21. BIBLIOGRAPHY (1 page maximum)

1. Massachusetts Department of Public Health. *Data Brief: Opioid-Related Overdose Deaths among Massachusetts Residents.* 2019.
2. Fauci AS, Redfield RR, Sigounas G, Weahkee MD, Giroir BP. Ending the HIV Epidemic: A Plan for the United States. *JAMA.* 2019;321(9):844-845.
3. Alpren C, Dawson EL, John B, Cranston K, Panneer N, Fukuda HD, Roosevelt K, Klevens RM, Bryant J, Peters PJ, Lyss SB, Switzer WM, Burrage A, Murray A, Agnew-Brune C, Stiles T, McClung P, Campbell EM, Breen C, Randall LM, Dasgupta S, Onofrey S, Bixler D, Hampton K, Jaeger JL, Hsu KK, Adih W, Callis B, Goldman LR, Danner SP, Jia H, Tumpney M, Board A, Brown C, DeMaria A, Jr., Buchacz K. Opioid Use Fueling HIV Transmission in an Urban Setting: An Outbreak of HIV Infection Among People Who Inject Drugs-Massachusetts, 2015-2018. *Am J Public Health.* 2020;110(1):37-44.
4. Cranston K, Alpren C, John B, Dawson E, Roosevelt K, Burrage A, Bryant J, Switzer WM, Breen C, Peters PJ. Notes from the Field: HIV Diagnoses Among Persons Who Inject Drugs—Northeastern Massachusetts, 2015–2018. *MMWR Morb Mortal Wkly Rep.* 2019;68(10):253.

5. Broz D, Carnes N, Chapin-Bardales J, Des Jarlais DC, Handanagic S, Jones CM, McClung RP, Asher AK. Syringe Services Programs' Role in Ending the HIV Epidemic in the U.S.: Why We Cannot Do It Without Them. *Am J Prev Med.* 2021;61(5 Suppl 1):S118-S129.
6. U.S. Centers for Disease Control and Prevention. *HIV Surveillance Report, 2018 (updated 2020).* 2020.
7. US Preventive Services Task Force. Preexposure Prophylaxis for the Prevention of HIV Infection: US Preventive Services Task Force Recommendation Statement. *JAMA.* 2019;321(22):2203-2213.
8. Choopanya K, Martin M, Suntharasamai P, Sangkum U, Mock PA, Leethochawalit M, Chiamwongpaet S, Kitisin P, Natrujirote P, Kittimunkong S, Chuachoowong R, Gvetadze RJ, McNicholl JM, Paxton LA, Curlin ME, Hendrix CW, Vanichseni S, Bangkok Tenofovir Study G. Antiretroviral prophylaxis for HIV infection in injecting drug users in Bangkok, Thailand (the Bangkok Tenofovir Study): a randomised, double-blind, placebo-controlled phase 3 trial. *Lancet.* 2013;381(9883):2083-2090.
9. Earlywine JJ, Bazzi AR, Biello KB, Klevens RM. High Prevalence of Indications for Pre-exposure Prophylaxis Among People Who Inject Drugs in Boston, Massachusetts. *Am J Prev Med.* 2020.
10. Centers for Disease Control and Prevention. *HIV Infection Risk, Prevention, and Testing Behaviors among Persons Who Inject Drugs—National HIV Behavioral Surveillance: Injection Drug Use, 23 U.S. Cities, 2018.* 2020.
11. Biancarelli DL, Biello KB, Childs E, Drainoni M, Salhaney P, Edeza A, Mimiaga MJ, Saitz R, Bazzi AR. Strategies used by people who inject drugs to avoid stigma in healthcare settings. *Drug Alcohol Depend.* 2019;198:80-86.
12. Motavalli D, Taylor JL, Childs E, Valente PK, Salhaney P, Olson J, Biancarelli DL, Edeza A, Earlywine JJ, Marshall BDL, Drainoni ML, Mimiaga MJ, Biello KB, Bazzi AR. "Health Is on the Back Burner:" Multilevel Barriers and Facilitators to Primary Care Among People Who Inject Drugs. *J Gen Intern Med.* 2020.
13. Biello KB, Bazzi AR, Vahey S, Harris M, Shaw L, Brody J. Delivering Preexposure Prophylaxis to People Who Use Drugs and Experience Homelessness, Boston, MA, 2018-2020. *Am J Public Health.* 2021;111(6):1045-1048.
14. Roth AM, Tran NK, Felsher M, Gadegbeku AB, Piecara B, Fox R, Krakower DS, Bellamy SL, Amico KR, Benitez JA, Van Der Pol B. Integrating HIV Preexposure Prophylaxis With Community-Based Syringe Services for Women Who Inject Drugs: Results From the Project SHE Demonstration Study. *Journal of acquired immune deficiency syndromes.* 2021;86(3):e61-e70.
15. Rowe C, Santos GM, Vittinghoff E, Wheeler E, Davidson P, Coffin PO. Predictors of participant engagement and naloxone utilization in a community-based naloxone distribution program. *Addiction.* 2015;110(8):1301-1310.
16. Lorvick J, Browne EN, Lambdin BH, Comfort M. Polydrug use patterns, risk behavior and unmet healthcare need in a community-based sample of women who use cocaine, heroin or methamphetamine. *Addict Behav.* 2018;85:94-99.
17. Lambdin BH, Comfort M, Kral AH, Lorvick J. Accumulation of jail incarceration and hardship, health status, and unmet health care need among women who use drugs. *Womens Health Issues.* 2018;28(5):470-475.
18. Sherr K, Gimbel S, Rustagi A, Nduati R, Cuembelo F, Farquhar C, Wasserheit J, Gloyd S, With input from the SST. Systems analysis and improvement to optimize pMTCT (SAIA): a cluster randomized trial. *Implement Sci.* 2014;9:55.
19. Gimbel S, Voss J, Mercer MA, Zierler B, Gloyd S, Coutinho Mde J, Floriano F, Cuembelo Mde F, Einberg J, Sherr K. The prevention of mother-to-child transmission of HIV cascade analysis tool: supporting health managers to improve facility-level service delivery. *BMC Res Notes.* 2014;7:743.
20. Rustagi AS, Gimbel S, Nduati R, Cuembelo Mde F, Wasserheit JN, Farquhar C, Gloyd S, Sherr K, with input from the SST. Implementation and Operational Research: Impact of a Systems Engineering Intervention on PMTCT Service Delivery in Cote d'Ivoire, Kenya, Mozambique: A Cluster Randomized Trial. *J Acquir Immune Defic Syndr.* 2016;72(3):e68-76.
21. Lambdin BH, Kral AH, Wagner AD, Wenger L, Sherr K. Optimizing Naloxone Distribution to Prevent Opioid Overdose Fatalities: Results from Piloting the Systems Analysis and Improvement Approach within Syringe Service Programs. *Dissemination and Implementation Research Conference (Oral Presentation; Virtual due to COVID).* 2020.
22. Bazzi AR, Biancarelli DL, Childs E, Drainoni ML, Edeza A, Salhaney P, Mimiaga MJ, Biello KB. Limited Knowledge and Mixed Interest in Pre-Exposure Prophylaxis for HIV Prevention Among People Who Inject Drugs. *AIDS Patient Care STDS.* 2018;32(12):529-537.
23. Biello KB, Bazzi AR, Mimiaga MJ, Biancarelli DL, Edeza A, Salhaney P, Childs E, Drainoni ML.

Perspectives on HIV pre-exposure prophylaxis (PrEP) utilization and related intervention needs among people who inject drugs. *Harm Reduct J.* 2018;15(1):55.
24. Biello KB, Mimiaga MJ, Valente PK, Saxena N, Bazzi AR. The Past, Present, and Future of PrEP implementation Among People Who Use Drugs. *Curr HIV/AIDS Rep.* 2021.
25. Brody JK, Taylor J, Biello K, Bazzi AR. Towards equity for people who inject drugs in HIV prevention drug trials. *Int J Drug Policy.* 2021:103284.
26. Earlywine JJ, Bazzi AR, Biello KB, Klevens RM. High Prevalence of Indications for Pre-exposure Prophylaxis Among People Who Inject Drugs in Boston, Massachusetts. *Am J Prev Med.* 2021;60(3):369-378.
27. Edeza A, Bazzi A, Salhaney P, Biancarelli D, Childs E, Mimiaga MJ, Drainoni ML, Biello K. HIV Pre-exposure Prophylaxis for People Who Inject Drugs: The Context of Co-occurring Injection- and Sexual-Related HIV Risk in the U.S. Northeast. *Subst Use Misuse.* 2020;55(4):525-533.
28. Felsher M, Ziegler E, Smith LR, Sherman SG, Amico KR, Fox R, Madden K, Roth AM. An Exploration of Pre-exposure Prophylaxis (PrEP) Initiation Among Women Who Inject Drugs. *Arch Sex Behav.* 2020;49(6):2205-2212.
29. North America Syringe Exchange Network. https://nasen.org. Published 2021. Accessed 8/31/21.
30. Lyss SB, Buchacz K, McClung RP, Asher A, Oster AM. Responding to Outbreaks of Human Immunodeficiency Virus Among Persons Who Inject Drugs—United States, 2016–2019: Perspectives on Recent Experience and Lessons Learned. *J Infect Dis.* 2020;222:239-249.
31. Ryerson AB, Schillie S, Barker LK, Kupronis BA, Wester C. Vital Signs: Newly Reported Acute and Chronic Hepatitis C Cases - United States, 2009-2018. *MMWR Morb Mortal Wkly Rep.* 2020;69(14):399-404.
32. Bazzi AR, Drainoni ML, Biancarelli DL, Hartman JJ, Mimiaga MJ, Mayer KH, Biello KB. Systematic review of HIV treatment adherence research among people who inject drugs in the United States and Canada: evidence to inform pre-exposure prophylaxis (PrEP) adherence interventions. *BMC Public Health.* 2019;19(1):31.
33. Roth AM, Goldshear JL, Martinez-Donate AP, Welles S, Chavis M, Van Der Pol B. Reducing Missed Opportunities: Pairing Sexually Transmitted Infection Screening With Syringe Exchange Services. *Sexually transmitted diseases.* 2016;43(11):706-708.
34. Roth A, Tran NK, Chavis M, Van Der Pol B. Examining unmet needs: a cross-sectional study exploring knowledge, attitudes and sexually transmitted infection screening preferences among persons who inject drugs in Camden, New Jersey. *Sex Transm Infect.* 2018;94(8):598-603.
35. Roth AMT, N. K.; Felsher, M. A.; Gadegbeku, A. B.; Piecara, B.; Krakower, D. S.; Bellamy, S. L.; Amico, R. K.; Benitez, J. A.; Van Der Pol, B. Integrating HIV pre-exposure prophylaxis with community-based syringe services for women who inject drugs: Results from the Project SHE demonstration study. *J Acquir Immune Defic Syndr.* 2020;available on-line first.
36. Felsher M, Ziegler E, Amico KR, Carrico A, Coleman J, Roth AM. “PrEP just isn't my priority”: Adherence challenges among women who inject drugs participating in a pre-exposure prophylaxis (PrEP) demonstration project in Philadelphia, PA USA. *Social Science & Medicine.* 2021;275:113809.
37. Felsher M, Ziegler E, Amico KR, Carrico A, Coleman J, Roth AM. "PrEP just isn't my priority": Adherence challenges among women who inject drugs participating in a pre-exposure prophylaxis (PrEP) demonstration project in Philadelphia, PA USA. *Soc Sci Med.* 2021;275:113809.
38. Tran NK, Felsher M, Pol BV, Bellamy SL, McKnight J, Roth AM. Intention to initiate and uptake of PrEP among women who injects drugs in a demonstration project: an application of the theory of planned behavior. *AIDS Care.* 2021;33(6):746-753.
39. Lambdin BH, Micek MA, Koepsell TD, Hughes JP, Sherr K, Pfeiffer J, Karagianis M, Lara J, Gloyd SS, Stergachis A. An assessment of the accuracy and availability of data in electronic patient tracking systems for patients receiving HIV treatment in central Mozambique. *BMC Health Serv Res.* 2012;12:30.
40. Gimbel S, Micek M, Lambdin B, Lara J, Karagianis M, Cuembelo F, Gloyd SS, Pfeiffer J, Sherr K. An assessment of routine primary care health information system data quality in Sofala Province, Mozambique. *Popul Health Metr.* 2011;9:12.
41. Lambdin BH, Micek MA, Koepsell TD, Hughes JP, Sherr K, Pfeiffer J, Karagianis M, Lara J, Gloyd SS, Stergachis A. Patient volume, human resource levels, and attrition from HIV treatment programs in central Mozambique. *J Acquir Immune Defic Syndr.* 2011;57(3):e33-39.
42. Pfeiffer J, Montoya P, Baptista AJ, Karagianis M, Pugas Mde M, Micek M, Johnson W, Sherr K, Gimbel S,

Baird S, Lambdin B, Gloyd S. Integration of HIV/AIDS services into African primary health care: lessons learned for health system strengthening in Mozambique - a case study. *J Int AIDS Soc.* 2010;13:3.
43. Lambdin BH, Cai T, Udoh I, Lu L, Lu X, Chang O, Plumley B. Identifying bottlenecks: loss to follow-up of MSM from HIV testing to treatment in Wuhan, China. Paper presented at: International AIDS Conference2012; Washington, DC.
44. Lambdin BH, Mbwambo JK, Josiah RM, Bruce RD. Service integration: Opportunities to expand access to antiretroviral therapy for people who inject drugs in Tanzania. *J Int AIDS Soc.* 2015;18:19936.
45. Tran OC, Bruce RD, Masao F, Ubuguyu O, Sabuni N, Mbwambo J, Lambdin BH. Implementation and operational research: linkage to care among methadone clients living with HIV in Dar es Salaam, Tanzania. *J Acquir Immune Defic Syndr.* 2015;69(2):e43-48.
46. Gupta A, Mbwambo J, Mteza I, Shenoi S, Lambdin B, Nyandindi C, Doula BI, Mfaume S, Bruce RD. Active case finding for tuberculosis among people who inject drugs on methadone treatment in Dar es Salaam, Tanzania. *Int J Tuberc Lung Dis.* 2014;18(7):793-798.
47. Lambdin BH, Masao F, Chang O, Kaduri P, Mbwambo J, Magimba A, Sabuni N, Bruce RD. Methadone treatment for HIV prevention-feasibility, retention, and predictors of attrition in Dar es Salaam, Tanzania: A retrospective cohort study. *Clin Infect Dis.* 2014;59(5):735-742.
48. Bruce RD, Lambdin B, Chang O, Masao F, Mbwambo J, Mteza I, Nyandindi C, Zamudio-Haas S, Buma D, Dunbar MS, Kilonzo G. Lessons from Tanzania on the integration of HIV and tuberculosis treatments into methadone assisted treatment. *Int J Drug Policy.* 2014;25(1):22-25.
49. Lambdin BH, Bruce RD, Chang O, Nyandindi C, Sabuni N, Zamudio-Haas S, McCurdy S, Masao F, Ivo Y, Msami A, Ubuguy O, Mbwambo J. Identifying programmatic gaps: Inequities in harm reduction service utilization among male and female drug users in Dar es Salaam, Tanzania. *PLoS One.* 2013;8(6):e67062.
50. Kim DD, Basu A, Duffy SQ, Zarkin G. The cost-effectiveness of treatments for individuals with alcohol use disorders. In: Neumann PJ, Sanders GD, Russell LB, Siegel JE, Ganiats TG, eds. *Cost-effectiveness in health medicine.* 2nd ed. New York, NY: Oxford University Press; 2017:385-430.
51. Scull TM, Kupersmidt JB, Malik CV, Morgan-Lopez AA. Using media literacy education for adolescent sexual health promotion in middle school: Randomized control trial of Media Aware. *Journal of Health Communication.* 2018;23(12):1051-1063.
52. Morgan-Lopez AA, Saavedra LM, Hien DA, Killeen TK, Back SE, Ruglass LM, Fitzpatrick S, Lopez-Castro T, Patock-Peckham JA. Estimation of equable scale scores and treatment outcomes from patient- and clinician-reported PTSD measures using item response theory calibration. *Psychol Assess.* 2020;32(4):321-335.
53. Greenfield SF, Sugarman DE, Freid CM, Bailey GL, Crisafulli MA, Kaufman JS, Wigderson S, Connery HS, Rodolico J, Morgan-Lopez AA, Fitzmaurice GM. Group therapy for women with substance use disorders: results from the Women's Recovery Group Study. *Drug Alcohol Depend.* 2014;142:245-253.
54. Morgan-Lopez AA, Fals-Stewart W. Analytic methods for modeling longitudinal data from rolling therapy groups with membership turnover. *Journal of Consulting and Clinical Psychology.* 2007;75(4):580.
55. Hien DA, Morgan-Lopez AA, Campbell AN, Saavedra LM, Wu E, Cohen L, Ruglass L, Nunes EV. Attendance and substance use outcomes for the Seeking Safety program: sometimes less is more. *J Consult Clin Psychol.* 2012;80(1):29-42.
56. Toth SL, Rogosch FA, Oshri A, Gravener-Davis J, Sturm R, Morgan-Lopez AA. The efficacy of interpersonal psychotherapy for depression among economically disadvantaged mothers. *Dev Psychopathol.* 2013;25(4 Pt 1):1065-1078.
57. Morgan-Lopez AA, Saavedra LM, Hien DA, Campbell AN, Wu E, Ruglass L. Synergy between seeking safety and twelve-step affiliation on substance use outcomes for women. *J Subst Abuse Treat.* 2013;45(2):179-189.
58. Payne J, Cluff L, Lang J, Matson-Koffman D, Morgan-Lopez A. Elements of a Workplace Culture of Health, Perceived Organizational Support for Health, and Lifestyle Risk. *Am J Health Promot.* 2018;32(7):1555-1567.
59. Morgan-Lopez AA, Elek E, Graham PW, Saavedra LM, Bradshaw M, Clarke T. A quasi-experimental evaluation of partnerships for success's impact on community-level ethanol and prescription drug poisoning rates. *Addict Behav.* 2019;95:220-225.
60. Morgan-Lopez AA, Saavedra LM, Hien DA, Campbell AN, Wu E, Ruglass L, Patock-Peckham JA, Bainter SC. Indirect effects of 12-session seeking safety on substance use outcomes: overall and attendance class-specific effects. *Am J Addict.* 2014;23(3):218-225.

61. Neumann PJ, Sanders GD, Russell LB, Siegel JE, Ganiats TG, eds. *Cost-effectiveness in health and medicine.* . United Kingdom: Oxford University Press; 2016.
62. Sanders GD, Neumann PJ, Basu A, Brock DW, Feeny D, Krahn M, Kuntz KM, Meltzer DO, Owens DK, Prosser LA, Salomon JA, Sculpher MJ, Trikalinos TA, Russell LB, Siegel JE, Ganiats TG. Recommendations for Conduct, Methodological Practices, and Reporting of Cost-effectiveness Analyses: Second Panel on Cost-Effectiveness in Health and Medicine. *JAMA.* 2016;316(10):1093-1103.
63. Lambdin BH, Bluthenthal RN, Wenger LD, Wheeler E, Garner B, Lakosky P, Kral AH. Overdose Education and Naloxone Distribution Within Syringe Service Programs - United States, 2019. *MMWR Morb Mortal Wkly Rep.* 2020;69(33):1117-1121.
64. Lambdin B, Bluthenthal R, Tookes HE, Wenger L, Lakosky P, Kral A. Understanding the impact of COVID-19 on the penetration of naloxone distribution from syringe service programs in the United States. *Dissemination and Implementation Research Conference (Oral Abstract; Virtual due to COVID).* 2021.
65. Lambdin B, Bluthenthal R, Tookes HE, Wenger L, Lakosky P, Kral A. Waiver of the Ryan Haight Act and Implementation of Buprenorphine Treatment at Syringe Service Programs. *Dissemination and Implementation Research Conference (Oral Abstract; Virtual due to COVID).* 2021.
66. Lambdin BH, Wenger L, Bluthenthal R, Garner B, LaKosky P, O'Neill S, Kral AH. Understanding the influence of external and internal characteristics on the penetration of naloxone delivery from syringe service programs in the United States. *Dissemination and Implementation Research Conference (Oral Presentation; Virtual due to COVID).* 2020.
67. Wenger LD, Kral AH, Bluthenthal RN, Morris T, Ongais L, Lambdin BH. Ingenuity and resiliency of syringe service programs on the front lines of the opioid overdose and COVID-19 crises. *Transl Res.* 2021;234:159-173.
